# GWAS markers in diagnostics of breast cancer risk

**DOI:** 10.1186/1897-4287-10-S4-A14

**Published:** 2012-12-10

**Authors:** Anna Jakubowska

**Affiliations:** 1 Pomeranian Medical University and Read Gene SA, Szczecin, Poland

## 

Breast cancer belongs to the most common malignancy diagnosed in women. The major inherited susceptibilities to breast cancer are germline mutations in mutations in high risk genes, eg. BRCA1, BRCA2, BRIP, PALB2, RAD51C, RAD51D (5-10%), mutations in moderate risk genes, eg. CHEK2, ATM, NBS1 (10-20%) which however, explain only a small number of breast cancer cases. The latter are alterations in low risk genes and other possible changes in genome, eg. constitutional methylations of BRCA1.

Genome – Wide Association Study (GWAS) was thought to help in identification of low risk alleles associated with the risk of particular disease. Up to know 14 GWAS conducted on Caucasian and 6 GWAS on Asian population (http://www.genome.gov/gwastudies/index.cfm?pageid=26525384#searchForm) have been performed on breast cancer cases and 21 variants associated with modest risk of BC (OR <1.3) have been identified.

In 2005 have been initiated Breast Cancer Association Consortium (BCAC), an international multidisciplinary forum of investigators interested in the inherited risk of breast cancer. The aim of the consortium is to combine data from many studies in order to identify genes/SNPs and provide a reliable assessment of the breast cancer risks. Currently the BCAC gathers 69 centers from 29 countries, including Poland, and has access to demographic, clinical and epidemiological data from over 76,000 breast cancer patients and 83,000 unaffected women. In GWAS performed by BCAC 14 SNPs have been identified to be associated with modest risks of breast cancer(per-allele ORs<1.3).

The aim of this study was to assess usefulness of SNPs identified in BCAC GWAS in diagnostics of breast cancer risk in Polish population.

We found that 6 out of 14 SNPs identified in BCAC GWAS were associated with breast cancer risk in Polish patients. Four additional SNPs have been found to be associated with moderate risk of breast cancer in Polish study. Overall, out of 10 SNPs strongest in Polish study 3 seem to be valuable markers for breast cancer diagnostics in Poland: OR 1.91, 95%CI 1.39-2.65, p 0.0001 for carriers of 2 risk alleles and OR 3.2, 95%CI 1.87-5.47, p<0.0001 for carriers of 3 risk allele.

